# Markov models of major depression for linking psychiatric epidemiology to clinical practice

**DOI:** 10.1186/1745-0179-1-2

**Published:** 2005-04-27

**Authors:** Scott B Patten

**Affiliations:** 1 Associate Professor, Dept. Community Health Sciences, University of Calgary. 3330 Hospital Drive N.W., Calgary, Alberta, Canada

**Keywords:** depressive disorder, major depression, epidemiology, cross-sectional studies, longitudinal studies.

## Abstract

**Background:**

Most epidemiological studies of major depression report estimates of period prevalence. Such estimates are useful for public health applications, but are not very helpful for informing clinical practice. Period prevalence is determined predominantly by incidence and episode duration, but it is difficult to connect these epidemiological concepts to clinical issues such as risk and prognosis. Incidence is important for primary and secondary prevention, and prognostic information is useful for clinical decision-making. The objective of this study was to decompose period prevalence data for major depression into its constituent elements, thereby enhancing the value of these estimates for clinical practice. Data from a series of population-based Canadian studies were used in the analysis. Markov models depicting incidence, prevalence and recovery from major depressive episodes were developed. Monte Carlo simulation was used to constrain model parameters to the epidemiological data.

**Results:**

The association of sex with major depression was found to be due to a higher incidence in women. In distinction, the higher prevalence in unmarried subjects was mostly due to a different prognosis. Age-related changes in prevalence were influenced by both factors. Education, which was not found to be associated with major depression in the survey data, had no impact either on risk or prognosis.

**Conclusion:**

The period prevalence of major depression is influenced both by incidence (risk) and episode duration (prognosis). Mathematical modeling of the underlying epidemiological relationships can make such data more readily interpretable in relation to clinical practice.

## Introduction

In recent decades, a large number of cross-sectional psychiatric epidemiological surveys have reported prevalence estimates for major depression. Prevalence data provide a clear perspective on the burden of major depression in the population, and these estimates are useful for health system priority setting and planning. Unfortunately, the implications for clinical practice are not as clearly evident. Sometimes, clinicians misinterpret prevalence estimates as estimates of risk, but this is an error because whereas incidence is a measure of risk, prevalence is influenced by episode duration (prognosis) and, to a lesser extent in the case of major depression, by mortality. Ideally, it would be possible to decompose associations that are observed in epidemiological prevalence data into their main determinants: incidence and episode duration.

Incidence reflects the probability of development of new depressive episodes. This parameter is particularly important for prevention. In primary prevention, an understanding of the risk of new episodes in sub-groups within the population can support the targeting of preventive efforts towards those groups at highest risk. Therapeutic decisions, such as those concerning the need for acute and maintenance-phase pharmacological treatment, depend upon an understanding of the expected course of a disorder in a particular patient An ability to decompose period prevalence data into more meaningful statements about risk and prognosis may increase the extent to which epidemiological data can inform clinical practice.

Some psychosocial factors that could plausibly be associated with major depression (e.g. education) have been found not to be associated with it in cross-sectional studies. In these instances, there continues to be some value in examining the ways in which incidence and prognosis combine to influence prevalence. A lack of association in cross-sectional data may be the end result of offsetting factors: an elevated incidence associated with improved prognosis, for example, may mask an important association in prevalence data. A psychosocial factor could influence prognosis in a clinically relevant way, even if that factor is not associated with major depression in cross-sectional epidemiological data.

In Canada, a National Study of Mental Health and Wellbeing has recently been conducted. This study utilized the WHO Mental Health 2000 version of the Composite International Diagnostic Interview (WMH CIDI) [1], and collected data from a nationally representative sample of 36,984 subjects. An expected pattern of cross-sectional association was observed (see Table 1). The prevalence was higher in women than in men, in young age categories, and in previously married subjects. There was no evidence of an association with education, a result that was also observed in the pan-European ESEMeD study [2]. Notably, an association with this variable was observed in the American National Comorbidity Survey [3] and its Replication [4]. Unfortunately, it is difficult to know whether the pattern of association with period prevalence represents differences in risk, or prognosis, or some intermixing of these factors.

**Table 1:** Cross-sectional Associations of Demographic Factors with Major Depressive Episode Prevalence*.

		12-month prevalence (%)	95% C.I.
Overall		4.8	4.5 – 5.1
			
Sex	Male	3.7	3.3 – 4.1
	Female	5.9	5.4 – 6.4
			
Age	15–25	6.2	5.4 – 7.0
	26–45	5.6	5.0 – 6.0
	46–65	4.4	3.8 – 5.0
	> 65	2.0	1.5 – 2.4
			
Marital Status	Wid/Sep/Div/Single	4.4	2.5 – 3.1
	Married/Single**	2.8	4.0 – 4.8
			
Education	Some > HS	4.8	4.4 – 5.3
	≤ High School	4.8	4.3 – 5.2

* Canadian Mental Health and Wellbeing Survey http://www.statcan.ca.

** includes "common law" status.

In this paper, Markov modeling procedures are used to synthesize several sources of epidemiological data from Canada, with the objective of recasting the data in a way that is more clinically useful.

## Methods

### 1. Markov modeling procedure

In a series of previous papers, a method for modeling the epidemiology of major depression has been described [5,6]. The method uses Markov models that simulate major depression epidemiology over a series of one week stages. The model contains two health states, depressed and non-depressed. Incidence is the transition probability associated with a change from the non-depressed to depressed state. The recovery pattern for major depression is more difficult to represent because, in major depression, the probability of transition between depressed and non-depressed states depends upon the amount of time spent in the depressed state (episode duration). As episodes become more chronic, the probability of recovery in any one week becomes smaller [7,8]. This reality makes the use of conventional epidemiological incidence-prevalence-mortality models such as the World Health Organization's DISMOD program [9] for major depression modeling somewhat questionable, although they do continue to be used for this purpose [10,11]. For these reasons, a "markov tunnel" [12] was used to model recovery in the analyses presented in this paper. At the onset of an episode, a subject enters the first stage of the tunnel: the first stage (or week) of the depressive episode. At the next stage, the subject either makes a transition back to the non-depressed state, or can move to the next stage in the tunnel (this is the complementary probability, such that the probability of one or the other of these events is 1.0). This next stage represents the second week spent in the interval, and so on. Using this procedure, the transition probabilities for recovery can be altered depending upon episode duration.

Preliminary work with this type of model has determined that the impact of mortality on major depression models is small [6]. In the current analyses, mortality was not considered and period prevalence is viewed as a composite measure reflecting incidence and prognosis. The transition probabilities for incidence and all of the weekly recovery probabilities (those transition probabilities associated with stages in the Markov tunnel) were linked to epidemiological data using Monte Carlo simulation [5]. Tracker variables were programmed to count the number of weeks spent in an episode, the proportion of simulated subjects developing episodes during intervals of time, etc. Possible values for the various transition probabilities were explored in order to find ones that predicted epidemiological parameter values similar to those estimated from suitable data sources.

### 2. Sources of data

This analysis utilized data from three different national survey projects. The first was a cross sectional study, the Canadian National Survey of Mental Health and Wellbeing http://www.statcan.ca/Daily/English/030903/d030903a.htm, which was a cross-sectional study that used the WHO Mental Health CIDI [1]. Incidence data derived from a national longitudinal study called the National Population Health Survey (NPHS) http://www.statcan.ca/english/concepts/nphs/nphs.htm. Episode duration data was derived from a large cross-sectional survey, the Canadian Community Health Survey (CCHS 1.1) http://stcwww.statcan.ca/english/sdds/3226.htm. The NPHS is a longitudinal study that follows a cohort of approximately 17,000 subjects with repeated interviews every two years. The study started in 1994 (interview process completed in 1995), and the interviews were repeated in 1996, 1998 and 2000. The CCHS 1.1 is a cross-sectional study with a sample size of n = 130,880 conducted in 2000. Both studies employed the CIDI Short Form for major depression (CIDI-SFMD) [13], which is a brief predictive interview that assesses 12-month prevalence of major depression. The positive predictive value of the CIDI-SFMD for CIDI-defined major depressive episode is approximately between 75% and 90% [13,14]. Using the NPHS, it is possible to estimate an approximation of annual incidence: the proportion of the cohort that were CIDI-SFMD negative at one interview (e.g. 1994), who were positive at their next interview two years later (in this case, 1996). Both studies included an item for those positive on the CIDI-SFMD, asking about weeks depressed in the past year. Data from the CCHS 1.1 was used in the analysis of this variable because the larger sample size led to greater precision of estimation.

While the incidence approximation in the NPHS has been used directly in some studies [15,16], Markov models can be used to refine these estimates [5]. The CIDI-SFMD includes an item that asks subjects classified as having major depression to report the number of weeks that they spent in the depressed state during the preceding year. The Markov modeling technique is useful for translating this weeks depressed in the past year data into a set of estimates for the weekly recovery rates for inclusion in a Markov tunnel [5]. Tracker variables are programmed into the Markov model to represent the probability of an episode in the last 52 weeks of a 104 week simulation interval (the incidence approximation available from the NPHS) and another tracker variable is programmed to count the number of weeks in the 52 weeks preceding an interview that were spent in the depressed state, simulating the data collected in the survey. Using Monte Carlo simulation, it is then possible to identify incidence rates and Markov tunnel recovery probabilities that are most consistent with the observed data [5,6].

In this project, these methods are employed in order to further delineate the cross-sectional associations observed in Table 1, assisting with a determination of whether these associations are due to differences in incidence or episode duration. For one variable, education (which was not found to be associated with major depression prevalence), the objective was to explore whether off-setting incidence-duration effects could explain this lack of association.

### 3. Data synthesis

In order to explore the epidemiology of major depression in relation to the categorical variables listed in Table 1, the NPHS and CCHS datasets were stratified by these variables. Marital status was divided into unmarried (divorced, widowed or separated, single) and married categories – with the latter category including "common law" relationships. Similarly, the subjects were divided into education and employment status categories. Because of the value of exploring multiple age levels, and the possibility of age-sex interactions, logistic regression models were created to explore the pattern of approximate incidence in these groups. An estimate of incidence was obtained in this way within the various strata for each of the three available NPHS cycles: 1994–96, 1996–98 and 1998–2000. Next, episode duration data for subjects within the specified age categories and for men and women were extracted from the CCHS 1.1 dataset. A series of Monte Carlo simulations were then run using ranges of possible values for incidence and recovery probabilities. The software, Data [17] was used for simulation. These trials were guided by a least squares minimization procedure to identify the set of transition probabilities that best explained the incidence and episode duration data [5].

## Results

The estimated incidence approximations, stratified by the potential explanatory variables are presented in Table 2. Since there was no evidence of variation across the data collection cycles, an average was taken and used in the Markov modeling simulations. These averages are presented in the right-hand column of Table 2. Generally, the incidence approximation follows a pattern resembling the prevalence pattern, except that marital status appears not to be associated with a difference in incidence.

**Table 2:** Major Depressive Episode, Approximation of Annual Incidence.

		Major Depressive Episode Incidence			
		1994–96	1996–98	1998–00	Average
Sex	Male	2.8 (1.9 – 3.6)	2.5 (1.9 – 3.1)	2.9 (2.2 – 3.6)	2.7
	Female	4.3 (3.7 – 5.0)	4.7 (3.8 – 5.5)	4.6 (3.8 – 5.3)	4.5
					
Age	12–25	5.3 (3.8 – 6.9)	5.2 (3.5 – 6.8)	4.8 (3.3 – 6.2)	5.1
	26–45	3.6 (2.7 – 4.5)	3.8 (3.1 – 4.5)	4.1 (3.6 – 5.2)	3.8
	46–65	2.8 (2.0 – 3.6)	2.8 (1.8 – 3.7)	2.7 (2.0 – 3.5)	2.8
	> 65	1.5 (0.5 – 2.4)	1.6 (0.6 – 2.6)	1.5 (0.6 – 2.4)	1.5
					
Marital Status	Wid/Sep/Div or Single	3.7 (2.4 – 5.0)	3.5 (2.4 – 4.5)	3.5 (2.5 – 4.5)	3.6
	Married*	3.6 (3.0 – 4.1)	3.5 (3.2 – 4.4)	3.8 (3.2 – 4.4)	3.6
					
Education	Some > HS	2.8 (2.2 – 3.2)	3.9 (3.2 – 4.7)	3.7 (3.1 – 4.4)	3.5
	≤ High School	4.6 (3.6 – 5.4)	2.9 (2.3 – 3.6)	3.8 (2.9 – 4.7)	3.8

* includes "common law" status.

### 1. Sex

**Figure 1:**
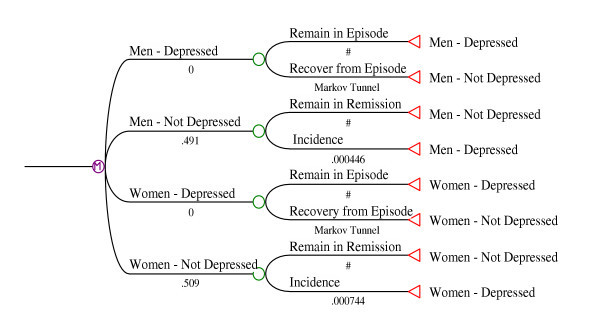
Markov Model for Major Depression, Stratified by Sex.

The reported number of weeks depressed in the past year were found to be almost identical for men and women. The same Markov tunnel was therefore used to simulate the pattern of recovery. The transition probabilities (TP) associated with each stage of the tunnel were found to be described adequately using the formula: TP_stage _= 0.14*e^-.047*stage^. This formula suggests that the probability of recovery in the initial week of an episode (ie. recovery by week three after the two week DSM-IV [18] duration criterion is met) is very high, approximately 14%. The weekly probability of recovery then declines by approximately 5% with each passing week. The final Markov model is depicted in Figure 1.

**Figure 2:**
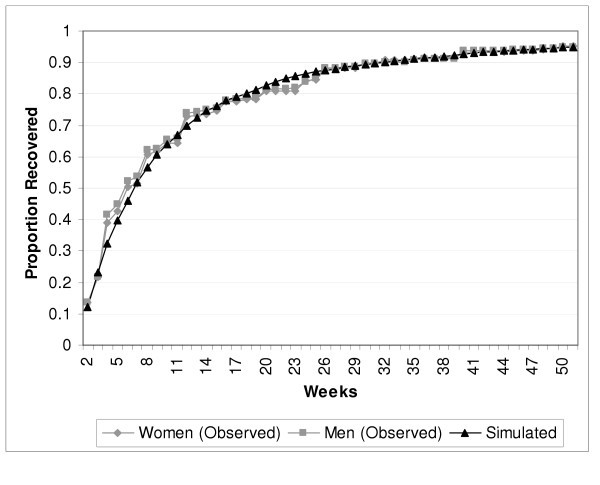
Observed and Simulated Episode Duration Data, by Sex.

Figure 2 presents the observed weeks depressed in the past year data from the CCHS (as a cumulative probability of recovery by week), and simulated values from the Markov model. Only one simulated curve is presented in the graphic because the curves for men and women were nearly identical. The simulated incidence approximation (the proportion of Monte Carlo simulations without an episode at baseline whose tracker variables indicate an episode in the last 52 weeks of a 104 week simulation run) varied linearly in relation to the incidence transition probabilities. The observed incidences (from Table 2) were predicted by the weekly transition probabilities depicted in Figure 1: 0.000446 in men and 0.000744 in women. The flattening of the curve with advancing weeks depicts the emergence of more chronic episodes in the sense that the recovery probabilities per week become quite small as an episode approaches one year in duration.

### 2. Age

**Figure 3:**
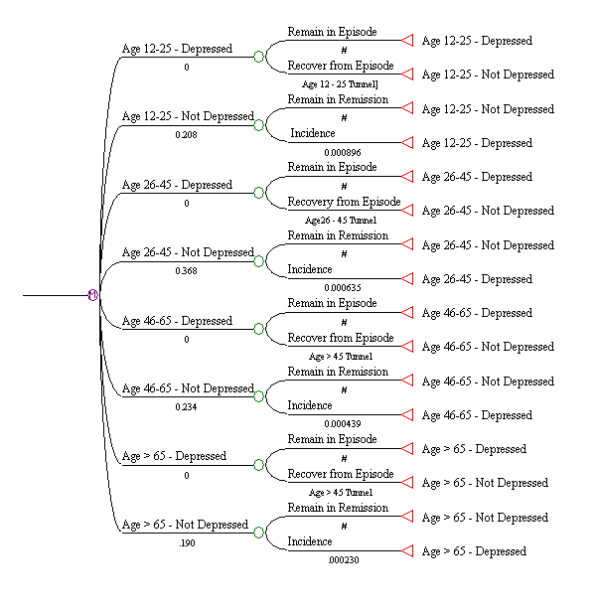
Markov Model for Major Depression, Stratified by Age Group.

**Figure 4:**
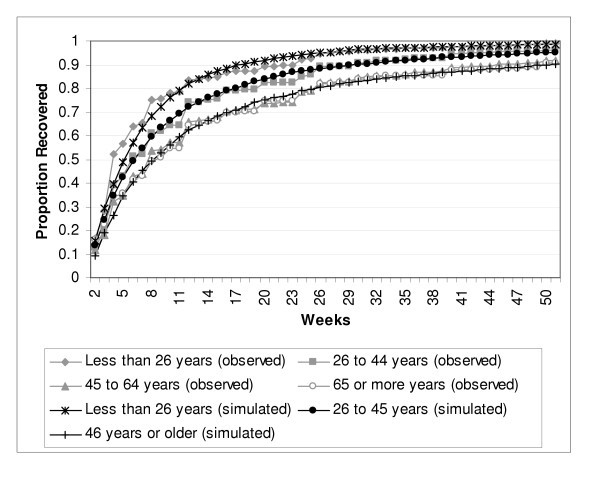
Observed and Simulated Episode Duration, by Age Group.

Age was evaluated at four levels, less than or equal to 25, 26–45, 46–65 and older than 65. The models suggested a more complex scenario than occurred for sex. In order to simulate the observed episode duration data, it was necessary to define three Markov tunnels, one for the less than or equal to age 25 (RP_group _= 0.19*e^-0.0443*week^), one for the 26 to 45 group (RP_group _= 0.15*e^-0.0499*week^) and one for the two older age categories, in other words, those over 45 years of age (RP_group _= 0.12*e^-0.0507*week^). The weekly incidence transition probabilities also needed to be higher in the younger age groups in order to reflect the age stratified incidence approximations estimated directly from the epidemiological data. The exponential parameters in the Markov tunnels indicate that the probability of recovery per week declines more quickly as the subjects' ages become larger. The final Markov model is depicted in Figure 3, and the observed and simulated duration data is presented in Figure 4.

### 3. Marital Status

**Figure 5:**
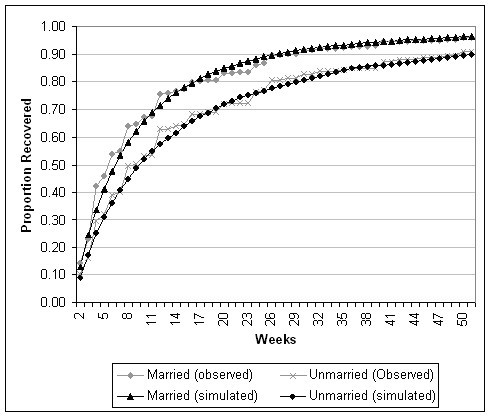
Observed and Simulated Episode Duration Data, by Marital Status.

The Markov model for marital status is not presented here, since its structure resembled that presented above for sex. As described above, marital status was analyzed at two levels. The estimated weekly incidence transition probability was similar in the married and single group (0.000537) and the previously married (0.000598) categories. However, different Markov tunnels were required to accurately simulate the episode duration data: RP_group _= 0.14*e^-0.0419*week ^for the married or single group and RP_group _= 0.10*e^-0.0461*week ^for the previously married group. A simulation of the episode duration data is presented in Figure 5.

### 4. Education

As seen in Table 1, major depression period prevalence was not associated with education level. It is possible that offsetting effects involving risk and prognosis could account for this. However, the Markov modeling did not suggest that this was the case. Simulation of the annual prevalence and episode duration data required only a single incidence probability and a single Markov tunnel for recovery. As such, the model did not differ from the unstratified model previously presented [5].

## Conclusion

The state of knowledge about major depression epidemiology is now supported by a large international literature of studies. However, the application of these data has largely been restricted to advocacy purposes and to broad-based priority setting exercises such as the global burden of disease project [10] or health economic studies [11,19,20]. Integrating period prevalence estimates with clinical practice is challenging. Traditionally, psychiatric assessment includes a formulation of etiological factors, and factors affecting prognosis are important for treatment planning, yet neither concept is closely linked to period prevalence.

Point prevalence is a more complex parameter than is often assumed, reflecting a steady state outcome of other factors. Period prevalence is even more complex. In this study, Markov models were used to synthesize various sources of epidemiological data, and to decompose these into estimates of parameters that are may be more useful to clinicians: those involving the risk of new episodes, episode prognosis, both of these factors or neither of them.

In the past, the most common application of Markov modeling in psychiatry has been in cost effectiveness analysis. Markov models are more widely used elsewhere in medicine to support clinical decision making, see review [12]. The simple models presented here help to clarify and synthesize epidemiological data in a way that could be integrated into clinical decisions concerning questions as the need for maintenance therapy, or the preferred duration of antidepressant treatment. In order to extend the approaches described in this paper towards application in quantitative decision analysis, it will be necessary to incorporate more variables (e.g. income, employment status, past history, family history) and also to incorporate procedures that account for more than one variable simultaneously.

Whenever modeling procedures are used to interpret empirical data, it is important to qualify the output based on the quality of the input data. Two of the three surveys used in this analysis incorporated only a predictive short form of the CIDI, and therefore may have been vulnerable to measurement bias. Measurement bias is probably the main threat to the validity of the data, as the samples were population based and good response rates were achieved (see survey documentation at http://www.statcan.ca). In addition to concerns that the CIDI-SFMD does not always agree with the full CIDI [14], the recent literature contains expressions of concern about the extent to which even the full CIDI can identify clinically significant episodes in the population [21,22].

In response to concern that episodes identified by the CIDI may not be clinically significant, some authors have suggested that epidemiological interviews should consistently incorporate clinical significance probes, typically eliciting subjects' descriptions of the severity and intrusiveness of their symptoms and their behavioral responses to their symptoms, e.g. seeking treatment [23]. Other authors have gone further, suggesting that interviewers in epidemiological studies should be trained to make relevant clinical judgments [21]. Brief instruments such as the CIDI-SFMD include neither clinical significance probes nor opportunities for clinical judgments to be made by the interviewers. However, to the extent that judgments about clinical significance are based upon assessment of factors affecting risk and prognosis, the approach described here offers certain empirical advantages. Rather than letting clinical judgment shape the collection of empirical data, they allow empirical clinical data an opportunity to shape and inform clinical judgment.

The demographic associations explored in this analysis are consistent with the current literature, providing some sense of confidence in their validity. Several previous studies have addressed the issue of an elevated prevalence in women by attempting to evaluate whether this is due to an effect on incidence or prognosis. Analyses deriving from the National Comorbidity Survey [24], the Vantaa Depression Study [25], the National Institute of Mental Health Collaborative Program on the Psychobiology of Depression [26] and the Baltimore Epidemiological Catchment Area Follow-up [27] have been consistent in observing a similar prognosis in men and women. This implies that the well-documented prevalence difference is probably due to incidence, the latter in fact having been confirmed by the Baltimore study [27]. The findings reported in this study are consistent with this literature.

Fewer studies have directly addressed the issue of age. The NCS did not include subjects over the age of 64. The Baltimore study, however, reported declining incidence with age [27], as reported here. In the Vantaa study, the univariate analysis showed a trend towards increasing time to full remission with age (p = 0.073). An analysis of data from the Epidemiological Catchment Area Study in the US also reported lower recovery rates after one year in older subjects [28], but the effect was largely confined to women.

The Baltimore study was consistent with the current findings in reporting no effect of education on episode duration [27]. However, the Baltimore study also reported that marital status had no "important" effect. In distinction to this, the current study found that an effect of unmarried status on prevalence was due to an impact of this variable on episode duration. An effect of marital status on prognosis seems plausible since many studies have found that personality disorders [25], and neuroticism scores [7] which would be expected to interfere with role functioning in relationships, are associated with diminished prognosis in major depression. However, marital status is likely to be a very crude indicator of these and a variety of other factors. More focused studies are required in order to more fully elucidate such effects.

In general, the pattern of recovery emerging from the Markov models developed in this analysis are comparable to the pattern reported by previous studies [7,25,29]. Markov models offer an interesting opportunity for integration of epidemiological data with clinical decision making.

## Competing Interests

The author(s) declare that they have no competing interests.
